# Visualisation of Respiratory Tumour Motion and Co-Moving Isodose Lines in the Context of Respiratory Gating, IMRT and Flattening-Filter-Free Beams

**DOI:** 10.1371/journal.pone.0053799

**Published:** 2013-01-10

**Authors:** Yvonne Dzierma, Frank G. Nuesken, Jochen Fleckenstein, Stephanie Kremp, Norbert P. Licht, Christian Ruebe

**Affiliations:** Department of Radiation Oncology, Saarland University Medical Centre, Homburg/Saar, Germany; Davidoff Center, Israel

## Abstract

Respiratory motion during percutaneous radiotherapy can be considered based on respiration-correlated computed tomography (4DCT). However, most treatment planning systems perform the dose calculation based on a single primary CT data set, even though cine mode displays may allow for a visualisation of the complete breathing cycle. This might create the mistaken impression that the dose distribution were independent of tumour motion. We present a movie visualisation technique with the aim to direct attention to the fact that the dose distribution migrates to some degree with the tumour and discuss consequences for gated treatment, IMRT plans and flattening-filter-free beams. This is a feasibility test for a visualisation of tumour and isodose motion. Ten respiratory phases are distinguished on the CT, and the dose distribution from a stationary IMRT plan is calculated on each phase, to be integrated into a movie of tumour and dose motion during breathing. For one example patient out of the sample of five lesions, the plan is compared with a gated treatment plan with respect to tumour coverage and lung sparing. The interplay-effect for small segments in the IMRT plan is estimated. While the high dose rate, together with the cone-shaped beam profile, makes the use of flattening-filter-free beams more problematic for conformal and IMRT treatment, it can be the option of choice if gated treatment is preferred. The different effects of respiratory motion, dose build-up and beam properties (segments and flatness) for gated vs. un-gated treatment can best be considered if planning is performed on the full 4DCT data set, which may be an incentive for future developments of treatment planning systems.

## Introduction

With the much improved conformality of state-of-the-art treatment planning, the need for positioning accuracy (e.g., [1]) has been increased to a point where respiratory motion becomes a notable issue to be considered in the treatment of lung tumours [2], [3], [4], [5], [6], [7], [8]. The development of four-dimensional respiratory-correlated computed tomography (4DCT) provides a means of creating a number of CT images correlated to particular breathing phases (e.g., [9]), which can be used for treatment planning and for the estimation of breathing effects on the treatment plan [10], [11]. In the simplest case, the GTV is delineated on all respiratory phases or the maximum/minimum intensity projection and then combined for treatment planning [12], [13], [14], [15]; more sophisticated approaches range from gated and breath-hold treatment [16], [17], [18], [19], [20], [21] to actual 4D treatment in which the beams are adapted to the respiratory phases or fractions, or track the tumour [22], [23], [24], [25], [26].

When considering the effect of tumour motion, it can be hypothesised that a solid tumour were moving through a fixed dose pattern. This notion was indeed adopted in early work as a simplification that could not be avoided at the time (compare, e.g., [27], [28]). However, in reality the dose distribution itself is influenced by the tumour motion due to dose build-up effects in the denser tumour tissue surrounded by low-density lung tissue, which means that the dose distribution will be changed by the tumour motion even when the treatment fields are fixed. Even though recent studies have pointed out that the interaction of the treatment fields with the tumour motion leads to a correlated motion of the isodoses with the tumour [29], [30], [31], [32], this is often not considered by the planning physicians due to shortcomings in the planning system, which usually do not allow to visualise the changing dose distribution over the range of tumour motion. This is mainly owing to the fact that treatment planning systems generally calculate the dose distribution based on one CT phase chosen as the “primary image set” (often the 3D “untagged” CT created without respiratory correlation), even if all respiratory phases can be displayed and animated as “secondary images”. While this “cine mode” display is unquestionably useful to estimate tumour motion for the validation of the combined GTV, it creates the mistaken impression that the tumour motion were independent of the dose distribution displayed alongside. The aim of this work is to present how the computation of the moving 4D dose distribution can be achieved and visualised for a better assessment of how much the isodose distribution is influenced by tumour motion and the respective advantages and disadvantages of

1. gated treatment vs. non-gated treatment
2. intensity-modulated radiotherapy (IMRT) vs. conformal radiotherapy (CRT)
3. flattening-filter-free (FFF) beams vs. flat beams.

## Patients and Methods

### Ethics Statement

All patients gave their written informed consent for 4D-CT examination and radiochemotherapy. An approval by the local ethics committee was not necessary due to the retrospective nature of this evaluation. The study was approved by the Institutional Review Board of the Radiotherapy Department of the Saarland University Medical Centre, chaired by Prof. Dr. M. Niewald, who issued a formal written waiver for the need of ethics approval. The research carried out here is in compliance with the declaration of Helsinki.

Image sets of 4DCT of four consecutive patients with five lesions who underwent stereotactic body radiotherapy (SBRT) of lung lesions were analysed to evaluate the dependency of dose distribution on tumour motion. Two of the lesions were treated with a fractionation of 5 times 12 Gy, three with 8 times 7.5 Gy (prescribed to the 80% isodose line); details on the patient and tumour characteristics are shown in Table 1.

**Table 1:** Baseline Demographic and Patient Characteristics.

Patient characteristics and Diagnosis	All patients (n = 4)
**Age, years (mean, range)**	68 (62–72)
**Sex** – **Male** **(no., %)**	4, 100
**Diagnosis – no. (%)**	
NSCLC^1^ (primary tumour)	2 (50)^2^
NSCLC (recurrent tumour)	1 (25)
Cancer of unknown primary (CUP)	1 (25)
**Localisation of lesions – no. (%)**	
right upper lobe	3 (60)
right lower lobe	2 (40)
**Dose and Fractionation – no. (%)**	
5×12 Gy (prescribed to 80% isodose line)	2 (40)
8×7.5 Gy (prescribed to 80% isodose line)	3 (60)

*Abbreviations:* 1) NSCLC = non-small cell lung cancer; 2) thereof one patient with two lung lesions.

All examined patients were immobilised by means of the dual vacuum BodyFIX® system (Medical Intelligence, Schwabmünchen, Germany). A 4D-spiral-CT (“slow-CT”) of the chest was obtained using a 16-slice CT scanner allowing respiratory correlated imaging (Brilliance CT Big Bore, Philips, Amsterdam, The Netherlands). For all patients, treatment plans for stereotactic body radiation therapy were created in the Philips Pinnacle treatment planning system (TPS) V.8 and V.9, for 6 MV photon fields and 7 MV flattening-filter-free fields for comparison.

4DCT was used to create a combined ITV based on the CT information from ten respiratory phases. The combined ITV was expanded by 0.5 cm to create the PTV, which encompasses the whole range of tumour motion plus a safety margin. Planning was carried out on the 3DCT data set obtained without respiratory correlation, so that the 80% isodose encompasses the whole ITV

To evaluate how the dose distribution created by the stationary plan optimised on the 3DCT changes over the respiratory cycle, the treatment plan was identically copied to all ten respiratory phase CT data sets (setting each phase CT as the primary data set) and recomputed. In this way, the plan is kept constant over the whole treatment, while the resulting dose distribution is determined for each respiratory phase

## Results and Discussion

The outlined method provides a simple way to assess the influence of tumour motion on the dose distribution. The information from the 4DCT is initially only used for the contouring of the ITV; the planning is carried out on the 3DCT without regard of further motion effects. After planning, the plan is copied to all 4DCT phases and the dose recomputed, which is a straightforward way to evaluate whether the plan quality is still acceptable during the full breathing cycle.

We demonstrate the visualisation technique for the patient with the largest isodose shift during the respiratory cycle. Tumour motion ranges between 0.2 and 1.3 cm for the five lesions analysed (Table 2). For the patient with the largest amplitude of tumour motion, the PTV is situated close to the dorsal chest wall, hence dose build-up effects inside the tumour are not pronounced and the dose distribution does not change markedly over the respiratory cycle. The strongest effect on the dose distribution is observed for patient 4, with the second largest tumour excursion (0.8 cm). For the other lesions, the dose distribution changes only slightly with tumour motion due to the small amount to tumour shift during breathing. We will therefore concentrate our discussion on patient 4 to give an example of the changing dose distribution for a case where tumour motion influences dose significantly.

**Table 2:** PTV and tumour motion characteristics.

Patient/lesion	Mean GTV/cm^3^	ITV/cm^3^	PTV/cm^3^	Maximum motion of tumour centre/cm
Patient 1 Ventral PTV	7.2	11.1	33.8	0.45
Patient 1 Dorsal PTV	4.5	9.9	31.3	1.28
Patient 2	4.3	7.0	24.3	0.44
Patient 3	2.5	4.4	17.7	0.24
Patient 4	10.1	15.5	45.0	0.80

Over the respiratory cycle, the volume of the GTV ranges between 9.5 cm^3^ and 10.76 cm^3^; the combined ITV has a volume of 15.48 cm^3^. For the treatment, an IMRT plan was chosen using 10 beams (6 MV) to achieve uniform coverage of the whole ITV inside the 80% isodose line.

If the cine mode display is used naïvely for the visualisation of the breathing effect, it would suggest that the tumour moved through a stationary dose distribution (Movie 1). This artefact is based on the fact that the dose distribution is normally only calculated on one CT primary image, in our case the 3DCT. If this display were used to assess the plan stability during the breathing cycle, it would create the mistaken impression that the tumour moved outside the high-dose region during the extreme respiratory phases. The real effect of tumour motion on the dose distribution, calculated on all respiratory phases, is shown in Movie 2 (created, as explained above, by calculating the isodose distribution of the identical plan on all 4DCT phases and combining them into a movie). Since the tumour stays within the open field throughout the respiratory cycle, dose build-up always occurs at the tumour, which is continuously followed by the isodoses and hence always well covered. The treatment plan therefore provides better tumour coverage than would be believed based on normal cine mode display.

### 1. What can be Gained by Gating?

For the case presented, the dose coverage follows the tumour over the whole respiratory cycle, so gating does not improve tumour coverage. It is generally observed that tumour coverage will not be improved by gating as long as the ITV is based on the full range of tumour motion, given the tumour is sufficiently small [31], [32], [33], [34]. If this is the case, the tumour will always stay within the beam during the full respiratory phase, and dose build-up will occur in the tumour. Hence, tumour control probability is not significantly compromised in the absence of gating.

Normal tissue complication probability may benefit from gating, (which can mean that increased doses may be prescribed for better tumour control; compare, e.g., [34]). Since only few respiratory phases are taken into consideration at the planning stage, the ITV becomes smaller and hence less dose is imparted to healthy tissue, in particular organs at risk. However, the amount of normal tissue sparing which could be achieved in our case is minor. We calculate and visualise a gated treatment plan for a 30% duty cycle (Movie 3). For this plan, the ITV volume is reduced from 15.5 cm^3^ (combined ITV) to 10.8 cm^3^ (gated), the corresponding PTV shrinks from 45.0 cm^3^ to 34.8 cm^3^. The dose to the lung is estimated by the V20 and V5 volumes (volumes of the lung receiving 20 Gy and 5 Gy dose, respectively) in Table 3. The values for the ipsilateral lung are virtually identical. For the contralateral lung, the V5 is reduced by almost 4%. The joint V5 for the total lung is reduced by less than 2%, while the V20 remains virtually unchanged. The most significant improvement occurs in the low dose region to the contralateral lung (V5); whether this is regarded as significant enough to perform gated treatment must be decided based on radiobiological considerations and tolerance to longer treatment times [32], [33], [35].

**Table 3:** Dose to lung for free-breathing and gated treatment plan.

		Free-breathing plan	Gated plan
Ipsilateral lung	V5	32.7%	31.6%
	V10	23.7%	22.1%
	V20	12.0%	11.5%
Contralateral lung	V5	11.0%	7.1%
	V10	0.8%	0.7%
	V20	0%	0%
Total lung	V5	22.7%	20.3%
	V10	13.2%	12.3%
	V20	6.5%	6.2%

This potential advantage of gating is counteracted by the disadvantage from the increase in treatment time. Usually, 20–40% of the respiratory cycle is used for gating, resulting in an increase in irradiation time by roughly a factor of three, which can complicate patient compliance. Even for patients who can tolerate the prolonged treatment time, the question arises whether intra-fraction motion (unrelated to breathing) may increase with treatment time and thus reduce the treatment accuracy gained by gating.

In conclusion, the trade-off between treatment time and normal tissue dose must be evaluated for every patient in the light of supportable treatment time and necessary/achievable normal tissue sparing. For this, it is important to take into account the changing dose distributions with respiration before deciding for or against gated treatment on an individual basis.

### 2. Interplay of MLC Motion with Tumour Motion in Free-breathing IMRT Treatment

**Figure 1:**
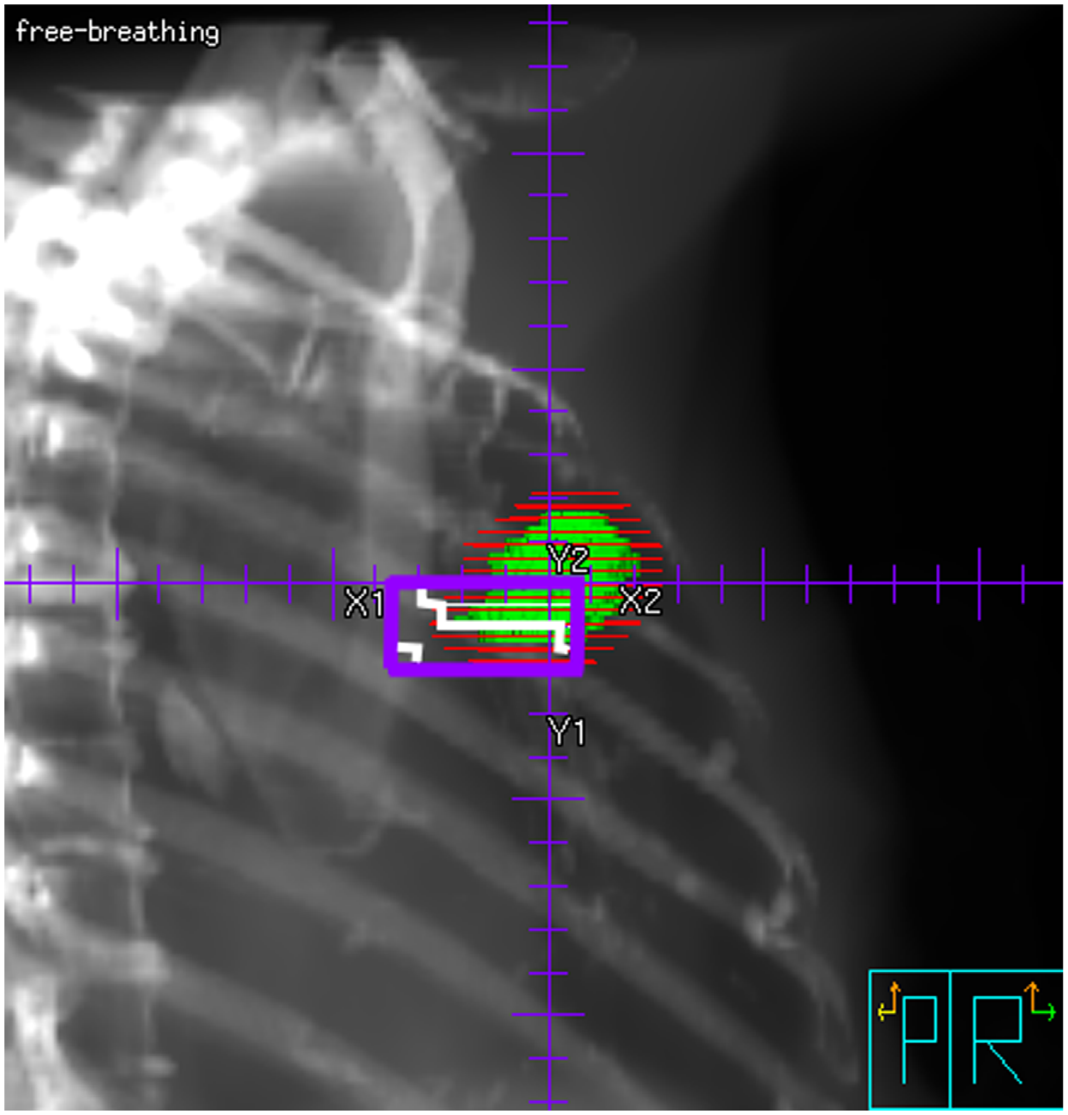
Example for a field segment with low monitor units (18 MU) located at the edge of the PTV. The segment is so small that the tumour may move completely outside in the respiratory phase (at the most cranial position). Due to the short irradiation time, this segment might hence miss the tumour. If this effect becomes significant for a number of fields, the dose distribution shown in Movie 2 is no longer valid, since it is based on the assumption that all respiratory phases will experience the same beam/segment configuration. More realistically, the low-MU segments will only be experienced by some respiratory phases. If the “wrong” phases are associated with the segments, the dose associated with these segments will not contribute to the PTV dose, but still increase the dose to organs at risk.

The case we consider uses an IMRT plan to achieve optimal tumour coverage with maximum sparing of the surrounding tissue. It is well-known that the interplay effect of the multi-leaf collimator (MLC) movement with the respiratory tumour motion can have detrimental effects on the dose coverage [7], [14], [36], [37]. This effect has only arisen with the introduction of IMRT technique, since traditional radiation plans using compensators to modulate the beam intensity did not rely on beam segmentation and reduced treatment times. In our case, all beams consisted of between two and five segments, of which at least one field segment (ca. 60–90% weight) irradiates the whole PTV with a small margin, and the remaining fields fill in small parts of the fluence where necessary. There are eight segments (out of a total of 34) with less than 20 MU, and three fields which are located at the edge of the PTV, i.e., in regions where the tumour is only encountered during a short time in the respiratory cycle. In this case, the dose motion together with the tumour no longer holds true, since the plan is not effectively stationary with respect to the tumour motion. Since 10 MU will require 2 s of irradiation with a dose rate of 300 MU/min, in the worst case, the tumour can be completely missed by these small segments (Figure 1). We have calculated the worst-case scenario by taking out the three segments which would miss the tumour); since the influence on the plan is minor, treatment was performed without accounting for this effect. However, if many such fields (with both small MU and small field-size at the edge of the PTV) were included in a treatment plan, the tumour motion could make the IMRT plan effectively worse than a stationary conformal plan.

There are two alternatives for coping with this difficulty in the case of un-gated, un-trailing treatment. The easiest method will be refraining from complicated IMRT plans and reverting to normal conformal treatment plans – however, this can only be an option in cases where the plan quality is not significantly impaired. As long as the IMRT plan is superior, a better choice will be to reduce the treatment dose rate for those segments with low MU. In our institution, the Siemens Artiste linear accelerator can be operated with a dose rate of either 300 MU/min or 50 MU/min for the 6 MV photon beam routinely used for IMRT. By using the low dose rate mode for those segments with 10–40 MU, the delivery time will be increased to at least 12s (10 MU), which spans more than two respiratory cycles (a similar idea, but for much higher dose rates and hence less pronounced effect, was presented by [38]). This latter approach will yield better plan quality, at the cost of somewhat increased treatment time. Compared with gated treatment, the increase is still relatively small, and may be a good compromise between gated treatment and standing open fields.

Neglecting the interplay effect may compromise the dose imparted to the tumour during one fraction; however, in fractionated therapy the importance of the effect diminishes. For each fraction, the initial respiratory phase at the start of treatment is arbitrary, hence the small-MU segments will be imparted during a different respiratory phase in each fraction, and the interplay effect will average out – considering the above example of a field irradiated during 2s, the total irradiation time over eight fractions will be 16 s, with a much greater probability of meeting all respiratory phases. The example presented in our study is rather a limiting case; the averaging effect over eight fractions is still not as good as would be achieved by a fractionation scheme with an increased number of fractions [39]. While the averaging effect over a number of fractions counteracts the interplay-effect and produces a comparably good PTV coverage in total, the dose difference between single fractions may be significant [24], [39]; whether this is biologically equivalent to a treatment plan with identical fractions is uncertain. A reduction in dose rate will hence be better suited for preventing the interplay-effect while creating a similar dose distribution in all fractions.

### 3. What is the Influence of Flattening-filter-free Beams?

We have seen that isodoses will follow the tumour location as long as the tumour stays within the beam, and will lead to a near-constant tumour dose if the tumour shape and volume do not change too much, and the beam profile is sufficiently flat. In the case of standing fields (CRT) with flattening-filter-free (FFF) beams, the cone-shaped dose profile will make the dose imparted to the tumour decrease with distance from the beam’s central axis as soon as the tumour motion from the axis exceeds several centimetres. Such large excursions are practically never the case in realistic clinical settings; for many practical purposes, the tumour motion will be of the order of at most 1–2 cm in either direction. However, the effect of this movement may become relevant if the tumour is not on the beam’s central axis, but in the region of the dose gradient of FFF beams. If CRT treatment is to be applied with FFF beams, it is hence of great importance to place the beam isocenter inside the tumour, since the beam intensity falls off laterally and the loss of flatness becomes significant for fields of more than ca. 5 cm width (compare, e.g., [40]). The dose imparted to the tumour will then be modulated by the changing beam intensity as a function of distance from the central axis, and the beneficial effect of the co-motion of isodoses and tumour will be reduced.

The cone-shaped beam profile is no longer a problem if the planning is performed in such a way to make the dose profile across the beam opening uniform (as is achieved with an IMRT plan). In this case, the resulting dose distribution will again have sufficient fluence at all tumour locations, and follow the tumour motion. For a FFF IMRT, however, a different complication arises from the higher dose-rate usually achieved for flattening-filter-free beams. The above discussed case of low-MU segments in IMRT plans cannot be solved easily for FFF beams. The Siemens Artiste 7XU beam at our institution can only be operated at dose rates of either 2000 MU/min or 500 MU/min. A reduction in dose rate to achieve “near-stationary” fields with respect to the tumour motion is hence not possible for low-MU segments (10 s would already correspond to 83 MU at the “low” FFF dose rate). The FFF beam should therefore not be used in these cases unless many fractions are applied to average out the interplay effect, as the inaccuracy of the treatment application is too high.

On the other hand, the increased dose rate available for FFF beams counteracts the protracted treatment time of a gated treatment. Given the fact that both CRT and IMRT are complicated for FFF beams, but the dose rate is drastically increased, it appears in conclusion that FFF beams should be the preferred option in the case that gating can be performed (compare, e.g., [41]), but flat beams should be preferred for IMRT and conformal treatment plans.

### Longitudinal Effects in dose Build-up

The presented example evaluates only one special case of dose build-up and co-motion with the tumour, where the tumour is moving laterally inside the open field. The direction of tumour motion will play an important role in the dose distribution, since the distance of the tumour from the linac head will influence the dose by the inverse square law. A purely longitudinal motion along the beam axis will produce a periodic variation in the dose due to the changing distance of the tumour from the accelerator head; however, the change in dose is minor as long as the tumour motion is within a 1–2 centimetres (which is virtually always the case).

If the tumour size exceeds a few centimetres, dose build-up at the tumour surface occurs together with dose decay with depth into the tumour; however, as along as the tumour does not change volume and shape, this effect will be similar over the whole respiratory cycle.

### Consequences for Future Developments of Treatment Planning Systems

It is vital that treatment planning systems be capable not only of including individual respiratory phase CTs as secondary data sets, but allow for dose calculation on all respiratory phases. Conventional practice of calculating the dose on the primary 3DCT and displaying this dose distribution on the “cine mode” respiratory CT phases will give to the wrong impression that the tumour will migrate out of the prescribed isodose region, which can be incorrect. Only an accurate dose calculation from the treatment plan on all 4DCT phases can enable the planning team to evaluate whether the proposed stationary treatment plan will cover the tumour sufficiently well with maximum sparing of surrounding tissues. Once this facility is provided by treatment planning systems, it can be decided for each patient where to place the limit between maximum tumour coverage and normal tissue irradiation. In particular, it can be tested in the TPS how far the ITV can be restricted to still achieve good tumour dose coverage, or whether gating might improve the treatment plan. Even where this is deemed unnecessary, a good evaluation of the plan quality will more reliably be achieved by considering the real breathing-induced effect on the dose distribution.

### Summary Discussion

Summarising the above discussion, the following main points have arisen:

- The dose build-up at the tumour means that the isodoses will move together with the tumour within the open field, resulting in good coverage in most clinical cases.
- The real dose behaviour and plan quality can only be assessed by looking at the dose computation on all breathing phases.
- Gating is only necessary in cases where the tumour motion is extreme (a few centimetres at least), so that the open field can be reduced to contain only a part of the tumour motion. In this case, it must be evaluated if this reduction can markedly reduce the dose to normal tissue, in particular lung dose.
- If gating is performed, flattening-filter-free beams provide a good option for counteracting the increase in treatment time and will provide good plan quality for gated IMRT.
- In non-gated IMRT treatment, the interplay effect is more exacerbated the higher the dose rate. Therefore, non-gated IMRT with small segments and low monitor units may benefit from reduced dose rate to reduce the interplay effect.
- In non-gated IMRT and conformal treatment, flat beams are generally preferable because the interplay-effect in IMRT is smaller and the conformal treatment often relies on flat fields so that dose build-up will be comparable everywhere within the field.

These statements are only meant as a rough guideline which summarises some general phenomena. For each individual patient case, they must be considered based on the dose movement over the respiratory cycle, for which calculation of the dose on the 4DCT breathing phases provides a good evaluation.

We must emphasise that these hypotheses are based on practical considerations of the underlying physics processes and are here demonstrated for a small number of patients. The conclusions hence suffer from the small sample size and should be validated in further studies. In particular, the benefit of using gated treatment for better lung sparing should be evaluated by means of planning studies, and with possible further clinical studies. The interplay effect, which has been considered in a number of studies, has neither been systematically assessed for flattening-filter-free beams nor for purposely reduced dose rates of flat beams so far. Finally, a more in-depth understanding of these effects and possible combinations of techniques will greatly benefit from a wider use of 4DCT single-phase calculated dose distributions at the treatment planning stage.

### Conclusions

We have presented a feasibility study of calculating the dose distribution on a 4DCT data set of 10 respiratory phases, for a stationary treatment plan created on the combined ITV (and optimised on the 3DCT data set). Even for the example case of significant tumour motion (0.8 cm), the tumour is well tracked by the isodoses during free-breathing – the plan is more conformal than would appear to be the case in “cine view” display without dose calculation on the separate breathing phases. Here, there is clearly no need for gated treatment. For those segments of the IMRT treatment plan with very low monitor units and small beam openings, the dose rate should be reduced so that the segment irradiation is prolonged to include a few respiratory cycles. If the treatment was performed with flattening-filter-free beams, the dose rate cannot be sufficiently reduced to make IMRT treatment a viable option. CRT can also be problematic due to the un-flat beam profile, which will not allow the isodose lines to follow the tumour motion as well as for flat beams. These two caveats, which apply only to FFF beams, are balanced by the increased dose rate, which can offset the increase in treatment time by gating and hence skews the decision towards FFF beams for gated treatment.

We suggest for the future development of treatment planning systems that a dose calculation with a given plan on each respiratory phase should be included, which will allow the planning team a more realistic evaluation of the plan quality and robustness.

## Supporting Information

Movie S1 **Appearance of tumour motion with isodoses as it is displayed in a typical TPS cine mode.** Here, the dose distribution is calculated on the primary data set, the “untagged” 3DCT without respiratory correlation. The same dose distribution is overlain on all respiratory phases. Attention – this is not the true dose distribution, since this would change with the respiratory phase (compare Movie 2). Isodoses are displayed as percentage of the dose at the reference point (75 Gy). At the most ventral position of the tumour, it appears to move outside the 95% isodose line, which is in fact not true. As will become clear in Movie 2, this effect is caused by the calculation on the 3DCT phase rather than the true respiratory phase.
(MPG)

Movie S2 **Tumour motion with true isodoses calculated on each respiratory phase separately.** The isocenter stays fixed at the same location and the beam configuration is unchanged – the dose movement is purely caused by the build-up effect at the tumour. Isodoses are displayed as percentage of the dose at the reference point (75 Gy). This is the “true” dose distribution over time as long as the treatment fields are stationary with respect to the moving tumour.
(MPG)

Movie S3 **Gated treatment plan with a 30% duty cycle.** The tumour motion over the duty cycle is negligible. Isodoses are displayed as percentage of the dose at the reference point (75 Gy).
(MPG)

## References

[1] Boda-Heggemann J, Lohr F, Wenz F, Flentje M, Guckenberger M (2011) kV Cone-Beam CT-Based IGRT. Strahlentherapie und Onkologie 5: 284–291. 10.1007/s00066-011-2236-4 21533757

[2] Ross CS, Hussey DH, Pennington EC, Standford W, Doornbos F (1990) Analysis of Movement of intrathoracic neoplasms using ultrafast computerized tomography. Int. J. Radiation Oncology Biol. Phys. 18: 671–677. 10.1016/0360-3016(90)90076-v 2318701

[3] Balter JM, Ten Haken RK, Lawrence TS, Lam KW, Robertson JM (1996) Uncertainties in CT-based radiation therapy treatment planning associated with patient breathing. Int. J. Radiation Oncology Biol. Phys. 36(1): 167–174. 10.1016/s0360-3016(96)00275-1 8823272

[4] Stromberg JS, Sharpe MB, Kim LH, Kini VR, Jaffray DA, et al (2000) Active breathing control (ABC) for Hodgkin’s disease: reduction in normal tissue irradiation with deep inspiration and implications for treatment. Int. J. Radiation Oncology Biol. Phys. 48(3): 797–806. 10.1016/s0360-3016(00)00681-7 11020577

[5] Langen KM, Jones DTL (2001) Organ motion and its management. Int. J. Radiation Oncology Biol. Phys. 50(1): 265–278. 10.1016/s0360-3016(01)01453-5 11316572

[6] Allen AM, Siracuse KM, Hayman JA, Balter JM (2004) Evaluation of the influence of breathing on the movement and modelling of lung tumours. Int. J. Radiation Oncology Biol. Phys. 58(4): 1251–1257. 10.1016/j.ijrobp.2003.09.081 15001270

[7] Webb S (2006) Motion effects in (intensity modulated) radiation therapy: a review. Phys. Med. Biol. 51: R403–425. 10.1088/0031-9155/51/13/R23 16790915

[8] Liu HH, Balter P, Tutt T, Choi B, Zhang Y, et al (2007) Assessing respiration-induced tumour motion and internal target volume using four-dimensional computed tomography for radiotherapy of lung cancer. Int. J. Radiation Oncology Biol. Phys. 68(2): 531–540. 10.1016/j.ijrobp.2006.12.066 17398035

[9] Sonke JJ, Zijp L, Remeijer P, van Herk M (2005) Respiratory correlated cone beam CT. Med. Phys. 32(4): 1176–1186. 10.1118/1.1869074 15895601

[10] Underberg RWM, Langerwaard FJ, Cuijipers JP, Slotman BJ, van Sörnsen de Koste JR, et al (2004) Four-dimensional CT scans for treatment planning in stereotactic radiotherapy for stage 1 lung cancer. Int. J. Radiation Oncology Biol. Phys. 60(4): 1283–1290. 10.1016/j.ijrobp.2004.07.665 15519801

[11] Frazier RC, Vicini FA, Sharpe MB, Yan D, Fayad J, et al (2004) Impact of breathing motion on whole breast radiotherapy: a dosimetric analysis using active breathing control. Int. J. Radiation Oncology Biol. Phys. 58(4): 1041–1047. 10.1016/j.ijrobp.2003.07.005 15001243

[12] Underberg RWM, Lagerwaard FJ, Slotman BJ, Cijipers JP, Senan S (2005) Benefit of respiration-gated stereotactic radiotherapy for stage 1 lung cancer: an analysis of 4DCT datasets. Int. J. Radiation Oncology Biol. Phys. 62(2): 554–560. 10.1016/j.ijrobp.2005.01.032 15890600

[13] Rietzel E, Liu AK, Doppke KP, Wolfgang JA, Chen AB, et al (2006) Design of 4D treatment planning target volumes. Int. J. Radiation Oncology Biol. Phys. 66(1): 287–295. 10.1016/j.ijrobp.2006.05.024 16904528

[14] Ding C, Li X, Saiful Huq M, Saw CB, Heron DE, et al (2007) The effect of respiratory cycle and radiation beam-on timing on the dose distribution of free-breathing breast treatment using dynamic IMRT. Med. Phys. 34(9): 3500. 10.1118/1.2760308 17926953

[15] Liu J, Wang JZ, Zhao JD, Xu ZY, Jiang GL (2012) Use of combined maximum and minimum intensity projections to determine internal target volume in 4-dimensional CT scans for hepatic malignancies. Radiation Oncology 7: 11. 22284745 10.1186/1748-717X-7-11 PMC3283494

[16] Korreman SS, Pedersen AN, Nøttrup TJ, Specht L, Nyström H (2005) Breathing adapted radiotherapy for breast cancer: Comparison of free breathing gating with the breath-hold technique. Radiotherapy and Oncology 76: 311–318. 16153728 10.1016/j.radonc.2005.07.009

[17] Saw CB, Brandner E, Selvaraj R, Chen H, Saiful Huy M, et al (2007) A review on the clinical implementation of respiratory-gated radiation therapy. Biomedical Imaging and Intervention Journal 3(1): e40. 21614265 10.2349/biij.3.1.e40 PMC3097646

[18] Zhao B, Yang Y, Li T, Li X, Heron DE, et al (2009) Image-guided respiratory-gated lung stereotactic body radiotherapy: Which target definition is optimal? Med. Phys. 36(6): 2248–2257. 10.1118/1.3129161 19610314

[19] Brock J, McNair HA, Panakis N, Symonds-Tayler R, Evans PM, et al (2011) The use of the active breathing coordinator throughout radical non-small-cell lung cancer (NSCLC) radiotherapy. Int. J. Radiation Oncology Biol. Phys. 81(2): 369–375. 10.1016/j.ijrobp.2010.05.038 20800379

[20] Gabrýs D, Kulik R, Trela K, Ślosarek K (2011) Dosimetric comparison of liver tumour radiotherapy in all respiratory phases and in one phase using 4DCT. Radiotherapy and Oncology 100: 360–364. 21974916 10.1016/j.radonc.2011.09.006

[21] Peguret N, Vock J, Vinh-Hung V, Fenoglietto P, Azria D, et al (2011) Breathing adapted radiotherapy: a 4D gating software for lung cancer. Radiation Oncology 6: 78. 21702952 10.1186/1748-717X-6-78 PMC3148530

[22] Keall PJ, Joshi S, Sastry Vedam S, Siebers JV, Kini VR, et al (2005) Four-dimensional radiotherapy planning for DMLC-based respiratory motion tracking. Med. Phys. 32(4): 942–951. 10.1118/1.1879152 15895577

[23] Alasti H, Cho YB, Vandermeer AD, Abbas A, Norrlinger B, et al (2006) A novel four-dimensional radiotherapy method for lung cancer: imaging, treatment planning and delivery. Phys. Med. Biol. 51: 3251–3267. 10.1088/0031-9155/51/12/017 16757875

[24] Webb S, Bortfeld T (2008) A new way of adapting IMRT delivery fraction-by-fraction to cater for variable intrafraction motion. Phys. Med. Biol. 53: 5177–5191. 10.1088/0031-9155/53/18/022 18728307

[25] Nohadani O, Seco J, Bortfeld T (2010) Motion management with phase-adapted 4D-optimization. Phys. Med. Biol. 55: 5189–5202. 10.1088/0031-9155/55/17/019 PMC3105327 20714043

[26] McQuaid D, Bortfeld T (2011) 4D planning over the full course of fractionation: assessment of the benefit of tumour trailing. Phys. Med. Biol. 56: 6935–6949. 10.1088/0031-9155/56/21/011 22008696

[27] Chui CS, Yorke E, Hong L (2003) The effects of intra-fraction organ motion on the delivery of intensity-modulated field with a multileaf collimator. Med. Phys. 30 (7): 1736–1746. 10.1118/1.1578771 12906191

[28] Vedam S, Docef A, Fix M, Murphy M, Keall P (2005) Dosimetric impact of geometric errors due to respiratory motion prediction on dynamic multileaf collimator-based four-dimensional radiation delivery. Med. Phys. 32(6): 1607–1620. 10.1118/1.1915017 16013720

[29] Rietzel E, Chen GTY, Choi NC, Willet CG (2005) Four-dimensional image-based treatment planning: target volume segmentation and dose calculation in the presence of respiratory motion. Int. J. Radiation Oncology Biol. Phys 61(5): 1535–1550. 10.1016/j.ijrobp.2004.11.037 15817360

[30] Flampouri S, Jiang SB, Sharp GC, Wolfgang J, Patel AA, et al (2006) Estimation of the delivered patient dose in lung IMRT treatment based on deformable registration of 4D-CT data and Monte Carlo simulations. Phys. Med. Biol. 51: 2763–2779. 10.1088/0031-9155/51/11/006 16723765

[31] Guckenberger M, Wilbert J, Krieger T, Richter A, Baier K, et al (2007) Four-dimensional treatment planning for stereotactic body radiotherapy. Int. J. Radiation Oncology Biology Physics 69(1): 276–285. 10.1016/j.ijrobp.2007.04.074 17707282

[32] Wu J, Li H, Shekhar R, Suntharalingam M, D’Souza W (2008) An evaluation of planning techniques for stereotactic body radiation therapy in lung tumours. Radiotherapy and Oncology 87: 35–43. 18359529 10.1016/j.radonc.2008.02.010 PMC2706126

[33] Seco J, Sharp GC, Wu Z, Gierga D, Buettner F, et al (2008) Dosimetric impact on motion in free-breathing and gated lung radiotherapy: A 4D Monte Carlo study of intrafraction and interfraction effects. Med. Phys. 35(1): 356–366. 10.1118/1.2821704 PMC2268084 18293590

[34] Roland T, Mavroidis P, Gutierrez A, Goytia V, Papanikolaou N (2009) A radiobiological analysis of the effect of 3D versus 4D image-based planning in lung cancer radiotherapy. Phys. Med. Biol. 54: 5509–5523. 10.1088/0031-9155/54/18/011 19717886

[35] Muirhead R, Featherstone C, Duffton A, Moore K, McNee S (2010) The potential clinical benefit of respiratory gated radiotherapy (RGRT) in non-small cell lung cancer (NSCLC). Radiotherapy and Oncology 95: 172–177. 20227779 10.1016/j.radonc.2010.02.002

[36] Bortfeld T, Jokivarsi K, Goitein M, Kung J, Jiang SB (2002) Effects of intra-fraction motion on IMRT dose delivery: statistical analysis and simulation. Phys. Med. Biol. 47: 2203–2220. 10.1088/0031-9155/47/13/302 12164582

[37] Chen H, Wu A, Brandner ED, Heron DE, Saiful Huy M, et al (2009) Dosimetric evaluations of the interplay effect in respiratory-gated intensity-modulated radiation therapy. Med. Phys. 36(3): 893–303. 10.1118/1.3070542 19378749

[38] Court L, Wagar M, Bogdanov M, Ionascu D, Schofield D, et al (2011) Use of reduced dose rate when treating moving tumours using dynamic IMRT. J. Appl. Clin. Med. Phys. 12(1): 258–34. 10.1120/jacmp.v12i1.3276 PMC5718590 21330973

[39] Jiang SB, Pope C, Al Jarrah KM, Kung JH, Bortfeld T, et al (2003) An experimental investigation of intra-fractional organ motion effects in lung IMRT treatments. Phys. Med. Biol. 48: 1773–1784. 10.1088/0031-9155/48/12/307 12870582

[40] Dzierma Y, Licht N, Nuesken F, Ruebe C (2012) Beam properties and stability of a flattening-filter free 7 MV beam – An overview. Med. Phys. 39(5): 2595–2602. 10.1118/1.3703835 22559630

[41] Mancosu P, Castiglioni S, Reggiori G, Catalano M, Alongi F, et al (2012) Stereotactic body radiation therapy for liver tumours using flattening filter free beam: dosimetric and technical considerations. Radiation Oncology 7: 16. 22296849 10.1186/1748-717X-7-16 PMC3292972

