# Correction: Visualisation of Respiratory Tumour Motion and Co-Moving Isodose Lines in the Context of Respiratory Gating, IMRT and Flattening-Filter-Free Beams

**DOI:** 10.1371/annotation/59fa459f-3ee2-4f53-8776-cd6b67131bcd

**Published:** 2013-11-04

**Authors:** Yvonne Dzierma, Frank G. Nuesken, Jochen Fleckenstein, Stephanie Kremp, Norbert P. Licht, Christian Ruebe

This article was republished on November 1, 2013 because of the release of confidential information. 

