# Correction: Visualisation of Respiratory Tumour Motion and Co-Moving Isodose Lines in the Context of Respiratory Gating, IMRT and Flattening-Filter-Free Beams

**DOI:** 10.1371/journal.pone.0094113

**Published:** 2014-03-28

**Authors:** The PLOS ONE Staff

**Notice of Republication**

This article was republished on March 7, 2014, due to the release of confidential or copyrighted information. Please download the article again to view the corrected version.
